# A Robotic Ultrasound System for Automated Abdominal Aorta Screening: Feasibility Study in Healthy Volunteers

**DOI:** 10.3390/s26144452

**Published:** 2026-07-13

**Authors:** Yixuan Zheng, Adam Geale, Philipp Kruse, Anoja Paraniroopasingam, Zhiyang Ma, Sarina Singh, Zhouyang Xu, Weizhao Wang, Yang Li, Shichao Zhang, Richard James Housden, Kawal Rhode

**Affiliations:** 1 School of Biomedical Engineering and Imaging Sciences, King’s College London, London SE1 7EH, UK; adam.t.geale@kcl.ac.uk (A.G.); philipp.kruse@kcl.ac.uk (P.K.); anoja.paraniroopasingam@kcl.ac.uk (A.P.); zhiyang.ma@kcl.ac.uk (Z.M.); zhouyang.xu@kcl.ac.uk (Z.X.); weizhao.wang@kcl.ac.uk (W.W.); yang.7.li@kcl.ac.uk (Y.L.); shichao.zhang@kcl.ac.uk (S.Z.); richard.housden@kcl.ac.uk (R.J.H.); kawal.rhode@kcl.ac.uk (K.R.); 2 Department of Biomedical Engineering, College of Engineering, University of Michigan, Ann Arbor, MI 48109, USA; sarinars@umich.edu

**Keywords:** autonomous robotic ultrasound, force control, image quality assessment, medical image segmentation, abdominal aortic aneurysm, ultrasound screening

## Abstract

Ultrasound is safe, portable, and relatively low cost, and robotic ultrasound research is expanding across many diagnostic applications. Within this context, abdominal aortic aneurysm (AAA) screening remains comparatively unexplored, with few systems reporting end-to-end autonomous scanning and clinician-validated evaluation in volunteers. We present a conditionally autonomous (Level-3) robotic ultrasound system in which the operator defines the region of interest and confirms the target force band, after which the robot performs surface-constrained abdominal sweeps under force control and automatically selects diagnostic frames and estimates aortic diameter without further manual interaction during scanning. The system combines RGB-depth-based patient-to-robot registration, hybrid position–force control with a low-cost force sensor, and a post-acquisition image-analysis pipeline comprising rule-based aorta localisation, a composite image quality assessment (IQA) metric, and a transfer-learned U-Net segmentation baseline. In a feasibility study on ten healthy volunteers spanning BMI 18.6–33 and diverse sex and skin-tone profiles, the robot maintained stable contact within the target force band in all sessions and produced aortic images rated diagnostically acceptable by clinicians in all participants. Automated diameter measurements showed a mean absolute difference of 1.45 mm relative to clinician reference values, with 9/10 cases within 3 mm and all within the 5 mm screening criterion. Volunteer questionnaires indicated high levels of comfort and trust in the system. These results demonstrate the feasibility of operator-supervised, force-aware robotic AAA scanning and highlight the potential of low-cost robotic ultrasound for wider automated vascular imaging.

## 1. Introduction

**Figure 1:**
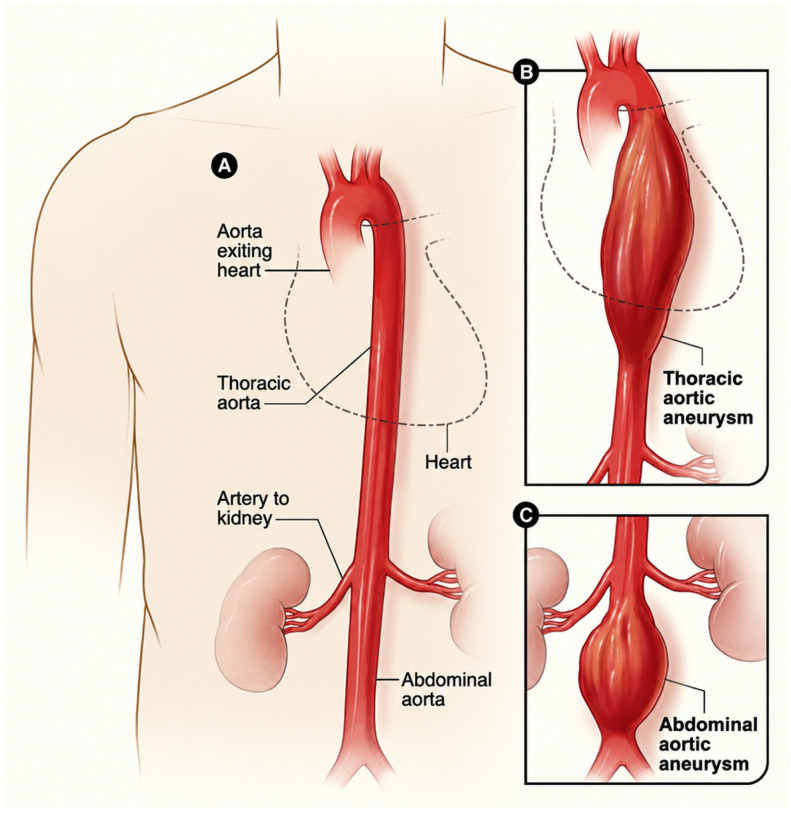
Anatomy of aortic aneurysms. (**A**) Normal thoracic and abdominal aorta. (**B**) Thoracic aortic aneurysm. (**C**) Infrarenal abdominal aortic aneurysm (AAA), which is the focus of this study [11].

An abdominal aortic aneurysm (AAA) is an abnormal bulging of the abdominal aorta, most often linked to ageing, smoking, hypertension, and genetic risk (Figure 1). Early detection is vital, as rupture is frequently fatal, and ultrasound (US) is the standard screening modality. US is radiation-free, safe for repeated use [1], real-time and portable [2], and comparatively low-cost [3], making it well suited for population-wide programmes. In the UK, for example, the NHS invites over 300,000 men for AAA scans annually; after a decade, rupture deaths have halved under structured internal quality assurance [4,5,6]. Nevertheless, diagnostic quality remains operator-dependent, with probe pose, contact force, and view selection introducing variability [7,8], while the workload contributes to ergonomic strain and infection-control risks [9,10]. These limitations motivate robotic assistance to improve consistency, reduce strain, and expand access to safe AAA scanning.

Robotic ultrasound (RUS) combines robotic positioning and compliant force control with image-driven planning to provide repeatable acquisitions and real-time quality checks under clinician oversight, with reported benefits for workflow reproducibility and service capacity [12,13]. Recent systems illustrate this trajectory under clinician oversight. For example, a fully autonomous thyroid pipeline [14], a standardised Auto-RUS workflow emphasising reproducibility [13], and an image-servoed carotid system [15].

To position our system, we adopt the levels-of-autonomy (LoA) framework for robotic ultrasonography proposed by Li et al. [16], in which higher levels correspond to reduced human involvement. Most reported prototypes cluster at Levels 1–2, where probe pose and contact are regulated automatically but anatomy interpretation remains operator-driven [16,17]. This work targets Level 3 (conditional autonomy), where perception, planning, and execution are integrated under clinician oversight; detailed comparisons with prior systems are deferred to Section 2.

Despite these advances, two gaps limit clinical deployment. First, in vivo validation remains sparse: aside from [14,18], most systems are evaluated on phantoms or only a handful of volunteers (1–3 subjects in [19,20,21]). Second, most approaches emphasise contact regulation without explicitly linking acquisition to diagnostic objectives; only a smaller body of work integrates downstream analysis for specific clinical tasks [14,15,18,21,22]. Complementary AAA-specific image-analysis and IQA literature [23,24,25,26,27,28] offers metrics that can inform RUS benchmarking but has not yet been coupled to end-to-end autonomous AAA acquisition. Our study addresses both gaps with a prospective feasibility study that links autonomy to standardised IQA and clinician-validated diameter estimation.

Specifically, this work makes the following contributions:

1. We design and implement a Level-3 (conditional-autonomy) robotic ultrasound (RUS) pipeline for AAA screening that integrates patient-to-robot registration, surface-constrained path planning, and hybrid position–force control into a single end-to-end workflow, enabling autonomous abdominal sweeps under clinician supervision once the operator has confirmed the scan region and target force band, with no manual probe manipulation thereafter.
2. We develop an aorta-centred, fully automatic, rule-based image-analysis pipeline that combines rule-based aorta localisation, a transparent composite image-quality metric for diagnostic frame ranking, and non-AI abdominal aorta diameter measurement, providing a lightweight and interpretable option suitable for early deployment.
3. We report a prospective feasibility study in ten healthy volunteers with diverse BMI, sex, and skin tone. In this cohort, the system consistently acquires diagnostic aortic views for all participants with scan durations comparable to manual examinations, maintains stable and safe contact forces, achieves automated diameter estimates in good agreement with clinician measurements, and is well accepted in post-scan questionnaires.
4. Learning-based benchmark: we implement a transfer-learned U-Net segmentation baseline on robotic ultrasound data and compare it with the proposed training-free, rule-based analysis for AAA screening, highlighting that competitive performance can be achieved without annotated training data.

The remainder of this paper is organised as follows. Section 2 reviews related work on robotic ultrasound and AAA image analysis. Section 3 describes the proposed robotic system, image-analysis pipeline, and volunteer study protocol. Section 4 presents the experimental results and discussion. Section 5 outlines future work, and Section 6 concludes the paper.

## 2. Related Work

### 2.1. Acquisition Foundations: Registration, Force Control, and Path Planning

Research on RUS commonly clusters around four themes [16]: (i) system registration and calibration; (ii) contact force control; (iii) scanning path planning; and (iv) ultrasound image analysis. Prior work, including our own, has addressed the first three areas; here we briefly summarise these foundations before focusing on image analysis, which underpins our Level-3 autonomous pipeline for AAA screening.

For registration, our earlier study used fiducial markers for patient-to-robot alignment and target tracking in extra-body imaging [29], while others have explored atlas- and vision-driven registration on articulated anatomy [30], confidence-based repositioning after motion [31], and dual-agent frameworks for spinal navigation [32].

In force control, we previously introduced a cost-effective force sensor and hybrid position–force controller [20]. Complementary work has demonstrated stable contact through integral controllers [33], reinforcement learning [34], inverse reinforcement learning for active compliance [19], and novel sensing modalities such as optical waveguides [35].

Path planning has evolved from anatomy-aware coverage with point-cloud guidance [20,36], to confidence-map–based orientation [37], to sample-efficient Bayesian optimisation for probe normalisation and image-quality-aware control [38,39], and even passive mechanisms for fetal scanning that encode safety by design [40,41]. These foundations reduce operator dependence while ensuring coverage and contact quality.

### 2.2. Ultrasound Image Analysis

Within RUS, image analysis closes the loop between perception and motion: visual feedback localises anatomy, evaluates view adequacy, and drives corrective probe actions. Fully autonomous pipelines have demonstrated this in thyroid scanning with end-to-end perception–planning–diagnosis [14] and in carotid imaging with image-driven servoing on human subjects [15,42]. Other systems apply confidence-map optimisation for alignment [37], real-time view classification or segmentation for quality-aware servoing [13,43], reinforcement-learning policies for standard-view navigation [32], deformation-aware vascular scanning [44], tubular-structure following [21], and force–ultrasound fusion [18]. High-level perception, however, requires reliable view-adequacy measures, a role filled by IQA.

Beyond anatomy recognition, reliable automation requires objective IQA rather than implicit proxies. On the AAA perception side, localisation has been addressed with geometry-driven methods such as variable neighbourhood search, which traces the aortic outer wall by minimising intensity and gradient costs [45], as well as deep learning on time-resolved 3D ultrasound to generate aortic and intraluminal thrombus (ILT) masks and compute diameters, though failures remain for small AAAs [23]. In robotic contexts, image feedback is often tied to contact: real-time image-based force adjustment and force–ultrasound fusion improve probe stability and view quality [12,18], while confidence-map optimisation and force-based probe positioning provide visual or force surrogates for alignment [37,46]. For quality auditing itself, phantom-based quality assessment has shown that contrast and Laplacian-based sharpness among a broader set of around 19 image metrics are particularly discriminative for classifying ultrasound image quality, with informative subsets identified through Linear Discriminant Analysis (LDA) [47]. This aligns with broader QA literature highlighting contrast/contrast-to-noise and edge/sharpness measures [27,28], and with clinical AI frameworks that approximate expert scoring in fetal ultrasound [48]. More recent learning-based ultrasound IQA approaches (including expert-agnostic deep variational clustering [49] and multiscale higher-order networks for bladder ultrasound [50]) report strong performance but typically require sizeable annotated datasets, which motivates the lighter-weight, training-free metric used in this work for early deployment. Evidence from spine ultrasound further shows that robotic and manual scans can match in reliability, motivating a common scorecard for AAA sweeps [51].

Compared to thyroid, cardiac, or fetal ultrasound, AAA-specific perception is relatively underexplored. The closest prior robotic AAA work [22] demonstrated force-compliant scanning with model-based registration, but evaluation was largely phantom-based and the system did not integrate automated frame-level quality assessment or end-to-end diameter estimation. Subsequent image-analysis studies have mainly focused on aortic segmentation and automated diameter estimation from B-mode or time-resolved 3D ultrasound [23,24,52], and in some cases, lumen and intraluminal thrombus delineation [25]. A recent prospective study also examined real-time deep learning guidance for novice AAA screening with frame-wise aorta localisation and post-scan diameter computation [26], yet these systems do not integrate quality gating into robot motion or undergo validation in settings that mimic robotic AAA screening. This gap motivates designs that (i) rank frames within each robotic sweep to retain a small set of diagnostically useful images, (ii) enable like-for-like quality comparisons between robotic and manual scans using the same objective scorecard, (iii) examine how contact force and visual quality covary across habitus and views, and (iv) detect the aorta to compute diameter automatically for downstream screening. In our study, these components are implemented to support autonomy decisions and to standardise evaluation across robotic and human acquisitions, with details provided in Section 3 and Section 4.

## 3. Materials and Methods

### 3.1. System Overview and Image Acquisition Workflow

#### 3.1.1. Hardware Setup

**Figure 2:**
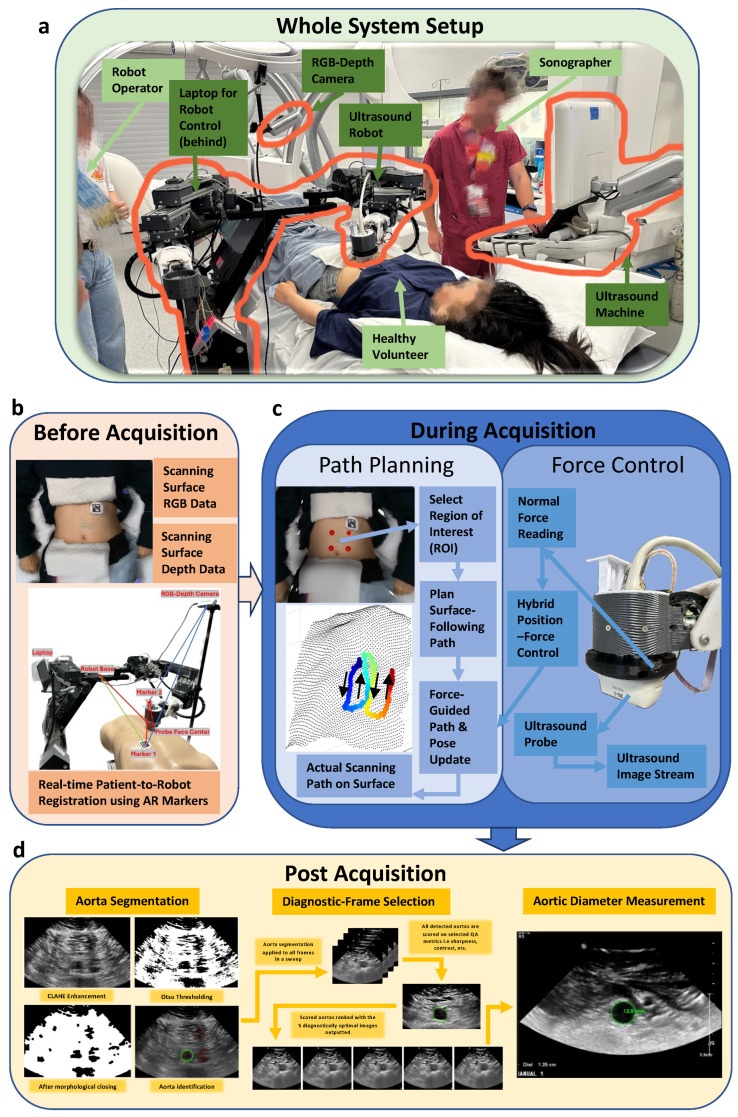
System workflow of the proposed robotic ultrasound platform. (**a**) Overall system setup, where dark green labels denote hardware components and light green labels denote human participants. (**b**) Pre-acquisition stage, including 3D patient surface generation and patient-to-robot registration. (**c**) Acquisition stage, robot generates surface-following path planning and uses hybrid position–force control to maintain probe contact. The robot adapts its scanning pose in real time based on force feedback. (**d**) Post-acquisition ultrasound image processing pipeline. Graphical key: in (**a**), red outlines delineate the main hardware components of the platform against the clinical background, and green arrows connect each label to the component it names. The large arrows between panels (**a**–**d**) indicate the order of the workflow. In (**c**), the colour-coded trajectory on the reconstructed abdominal surface is the planned scanning path, with black arrows showing the direction of the sweep, and the blue arrows denote the flow of data and control signals between processing steps. In (**d**), the yellow arrows denote the order of the post-acquisition processing steps; its ultrasound frames are the genuine cropped algorithm inputs and outputs, so fragments of console text remain at their edges (Section 3.2.4).

As shown in Figure 2a, we reuse our established dual-arm robotic ultrasound platform originally developed by previous research team for fetal screening [41]. Each arm provides eight-degrees-of-freedom (eight-DOF) for flexible probe placement and terminates in a wrist-mimicking probe holder for fine pitch/roll alignment. From left to right in the figure: the robot operator stands by the host laptop running the controller and real-time 3D visualisation; an Intel^®^ RealSense™  RGB–D camera (Intel Corporation, 2200 Mission College Blvd., Santa Clara, CA 95054, USA) observes the workspace at the centre, one robotic arm positions a clinical probe on the volunteer; and to the right, a clinician supervises the procedure at a standard ultrasound console (Philips EPIQ7G, Philips Healthcare, Bothell, WA, USA) with an X6-1 abdominal probe (1–6 MHz). All scans used the Philips Abdominal General (Abd Gen) preset on the EPIQ 7G with the X6-1 broadband probe (1–6 MHz), acquired at a display frame rate of 30 Hz and with low acoustic output (mechanical index MI =1.1; soft-tissue thermal index TIS =0.1). Importantly, the preset was optimised once by an experienced sonographer at the start of the study and then held constant across both manual and robotic acquisitions and across all volunteers, so that the image-quality scores are not confounded by changing acquisition settings. The only per-participant adjustment was imaging depth, set according to body habitus: typically about 5–11 cm for normal-weight volunteers (e.g., 5.1 cm for a slim manual reference and 8.1 cm for a robotic sweep in the examples analysed here) and increased to approximately 15–20 cm for the single highest-BMI participant (BMI =33) to reach the deeper aorta. No other acquisition or display parameter, including gain and dynamic range, was changed between scans or volunteers; gain and dynamic range are not embedded in the exported DICOM frames but were left at their fixed preset values throughout. The one scan acquired with a non-matching preset was excluded from the quantitative comparison (Section 4). The DICOM spatial calibration was isotropic ($s_{x}=s_{y}$), consistent with the scan-converted sector format, with the pixel spacing scaling with imaging depth (e.g., ≈0.10 mm/pixel at a 5 cm display depth and ≈0.14 mm/pixel at 8 cm); this measured isotropy is precisely why the orientation-dependent factor in Equation (2) reduces to a single spacing *s*.

The probe is mounted in a wrist-like end-effector that houses a lightweight, single-axis, 3D-printed force ring surrounding the probe [20]. A Teensy 4.1 acquires the load-cell signal and streams force to the controller. The force-sensing subsystem, characterised in our prior work [20], comprises four TE Connectivity FX29 compact compression load cells (model FX29K0-100A-0010-L, TE Connectivity, Berwyn, PA, USA; 10 lbf ≈ 44.5 N each) with digital I^2^C output; their readings are summed to give the axial contact force, so the combined measurement range is approximately 0–178 N, well above the contact forces used in this study. As reported in [20], the summed sensor was linear over the tested 0–34 N range ($R^{2}=0.9994$), with an accuracy of 0.1–0.69% of reading, a resting-noise standard deviation (effective resolution) of about 0.006 N, and agreement with a reference ATI Mini40 force/torque sensor at a root-mean-square error of 0.71 N.

For registration and patient tracking, an Intel RealSense RGB–D camera detects AprilTag markers [53] affixed to the robot end-effector and the patient’s abdomen, providing continuous patient-to-robot alignment. The AprilTag-based patient-to-robot registration follows the fiducial-marker method characterised in our earlier study [29], where, over 70 target poses, it achieved a mean in-plane (xy) positioning error of 5.5 mm (standard deviation 2.5 mm) and a mean depth error of 8.2 mm (standard deviation 5.3 mm) between the probe-face centre and the patient surface. A residual positioning offset of this magnitude sets only the initial probe placement; it is subsequently absorbed by the surface-following path planning and force control, which regulate contact during the sweep. This hardware stack, robot, probe holder, force sensor, RGB–D camera with AprilTag markers, and host PC, follows our prior publications [20,29].

Consistent with the low-cost motivation of this work, both the dual-arm manipulator and the force sensor are custom, in-house designs rather than commercial units. Their cost is therefore limited to inexpensive parts: load cells, 3D-printed components, machined metal parts, standard motors, and a microcontroller board (Teensy 4.1). This avoids the two most expensive elements of comparable robotic-ultrasound systems, namely a commercial force/torque sensor and an intrinsically force-controlled robotic arm, and patient-to-robot registration likewise relies only on a low-cost RGB–D camera with fiducial markers. The ultrasound console lies outside the system-cost boundary: because the probe holder is 3D-printed and can be re-modelled for any probe geometry, the platform is agnostic to the make of ultrasound machine.

#### 3.1.2. Software and Programming Workflow

A single C++ based application integrates robot control, compliant force regulation, path planning, and system registration, with a VTK-based 3D simulation view for supervision. Marker tracking uses the Open Source Computer Vision Library (OpenCV v4.7.0) to detect AprilTag markers on the end-effector and patient. At runtime, the pipeline has three phases: before acquisition, during acquisition, and post-acquisition (Figure 2b–d).

Before acquisition (Figure 2b), the Intel^®^ RealSense™ RGB–D camera captures the patient surface, from which a 3D mesh is reconstructed, smoothed, and used to compute surface normals. AprilTag markers on the robot end-effector and patient abdomen are then detected to solve the patient-to-robot transformation. This registration places the reconstructed surface into the robot base coordinate frame, ensuring that the visualisation and physical scene are aligned and providing the geometric basis for subsequent ROI selection and surface-following path planning.

During acquisition (Figure 2c), the operator first selects a region of interest (ROI) by dragging a rectangular selection over the reconstructed patient surface. The planner then generates an initial surface-following path within this region and projects the waypoints onto the post-processed 3D body mesh. The mesh is smoothed and hole-filled to ensure stable surface normals and to prevent depth noise from causing abrupt geometric changes. Hybrid position–force control maintains the desired Z-axis (normal) force while allowing tangential motion along the surface. When the measured force or probe pose deviates from the planned surface-following trajectory, the controller computes a corrective motion along the local surface normal and updates the probe pose and local path in real time. Throughout each sweep, the ultrasound probe acquires a continuous B-mode image stream, while force, pose, and image data are time-synchronised and logged.

Post-acquisition processing (Figure 2d) begins with frame-wise preprocessing and segmentation. Each ultrasound frame is first contrast-enhanced using CLAHE and then converted to a binary mask using Otsu thresholding, followed by morphological closing to fill small gaps and suppress speckle-induced holes. Candidate vessel regions are extracted from the mask and the abdominal aorta is identified based on geometric consistency. This segmentation is applied across all frames acquired during the sweep. A quantitative QA metric is then computed per frame to rank view adequacy, and the top-*k* diagnostic frames are selected. Finally, aortic diameter is estimated from the segmented lumen on the selected frames (green overlay), producing the post-acquisition measurement output. In addition to this training-free pipeline, we also evaluate a transfer-learned U-Net segmentation baseline offline for comparison (Section 3.3).

### 3.2. Rule-Based Post-Acquisition Analysis

#### 3.2.1. Rule-Based Aorta Localisation

In B-mode abdominal vascular ultrasound, the blood-filled aortic lumen appeared as a dark (hypoechoic) region surrounded by a brighter echogenic wall. Given the diagnostic significance of the infrarenal aortic segment, the pipeline’s first stage involved automatic aorta detection and segmentation to ensure consistent localisation across robotic sweeps.

The input consisted of B-mode ultrasound multi-frame DICOM files collected using the robotic system. Each frame was pulled out and reformatted to a 2D grayscale NumPy array using the pydicom library to get consistent pixel intensity structure for individual analysis. Preprocessing was started with Contrast Limited Adaptive Histogram Equalisation (CLAHE) enhancement and Gaussian Blurring (kernel size: 5×5) using a low-pass filter to attenuate high-frequency content and reduce ultrasound speckle without sacrificing low-frequency anatomical details such as vessel contours. This was followed by Otsu’s method of determining an optimal threshold which best separated the variance of foreground and background intensity distributions. Morphological closing using a 16 × 16 circular kernel removed small discontinuities and reconnected partially segmented vessel boundaries. All image processing kernel parameters were determined during pilot testing by iteratively adjusting a large range of values until clinically approved optimal aorta detection was consistently achieved across representative frames.

With the OpenCV library, all contours were extracted and subsequently filtered based on geometric criteria such as size, position, aspect ratio and spatial consistency to ensure the identification of near circular, stable regions consistent with aorta morphology. For qualifying contours, an expanded bounding box was drawn to include surrounding boundaries, as seen in Figure 2d with data extracted to assist diameter measurements and mask generation to isolate aortic regions for QA metric computation. Frame relevance checks were developed with the discarding of frames from the QA scoring pipeline if no aortic region was detected after filtering. Additionally, the generated mask was checked for internal artefacts using pixel intensity analysis to ensure retainment of diagnostically usable frames. This module provided a consistent aortic region for frame-level diagnostic ranking.

The following subsection builds on this automatic aorta localisation output and defines the composite image quality metric computed within the detected aortic region.

#### 3.2.2. Composite Image Quality Metric

From a deployment perspective, we deliberately prioritised a rule-based formulation for image-quality assessment rather than an end-to-end deep-learning predictor. In the AAA screening context, the core pipeline should remain robust even when only small, site-specific datasets were available and computational resources were limited. The composite metric relied solely on automatically derived aortic regions and simple pixel-wise operations on each DICOM frame. This enabled near-real-time execution on standard CPUs and made the scoring behaviour easy to inspect and tune in collaboration with clinicians.

In robotic ultrasound, continuous sweep acquisitions produce large frame volumes, increasing the burden on clinicians to review and select diagnostically relevant images. This module builds on the pipeline’s initial stage of aorta detection and segmentation to identify high-quality, diagnostically relevant frames while discarding artefact-heavy ones, thereby supporting reliable AAA measurements and reducing the risk of misinterpretation. For both manual and robotic acquisitions, the input to the QA module consists of individual frames together with the cropped aorta mask produced by the rule-based segmentation described in Section 3.2.

Within this aortic region, each frame is evaluated using three region-specific metrics: sharpness *S* (Laplacian variance) [54], grey-level dispersion *D* [55], and edge density *E* [56]. We use the term “grey-level dispersion” rather than “contrast” because *D* is the standard deviation of pixel intensities within the aortic region, i.e., a measure of local intensity spread rather than the target-to-background contrast familiar to ultrasound readers. It correlates with perceived image quality in phantom studies [47] but is not a normalised contrast measure and can be inflated by increasing display gain. A more rigorous, gain-invariant alternative is the generalised contrast-to-noise ratio (gCNR) [57], which needs a defined lumen target and a wall/tissue background; because our localisation already isolates the lumen and a surrounding ring, gCNR is a natural extension that we note as future work. To limit the influence of gain in this study, all frames within a given manual-versus-robotic comparison used the same imaging preset, and the single scan acquired with a divergent preset was excluded (Section 4). Under such a fixed display setting, the remaining variation in *D* is not driven by gain but by genuine acquisition-quality differences—in particular whether the probe is held normal to the surface and whether the contact force is sufficient to resolve the vessel wall, both of which sharpen the lumen–wall boundary and raise *D*. Within our fixed-preset acquisitions, *D* therefore remains a meaningful cue for ranking frames; adopting gCNR would additionally remove any residual dependence on display settings and enable comparison across different presets, which is why we highlight it as a worthwhile refinement rather than a change required for the present within-setting ranking. This handcrafted fusion metric provides a transparent and modular foundation for early deployment, allowing rapid validation, better alignment with clinical decision-making thresholds, and consistent performance across varied imaging conditions.

Each pre-processed frame containing a valid aorta mask is iteratively passed through the metric-computation function, with numerical scores calculated as pixel-wise operations restricted to the aortic region, as detailed in Algorithm 1. Each component metric (*S*, *D*, *E*) is min–max normalised to the range [0,1] across the pooled set of manual and robotic frames; manual and robotic frames are therefore scaled jointly, so that their composite scores are directly comparable on a common scale, and a weighted-sum fusion (Equation (1)) is applied to reflect metric sensitivity and diagnostic priority. Because the fusion weights sum to one, the normalised composite lies in [0,1]; for readability, all composite QA scores are reported on a 0–100 scale (i.e., the [0,1] value multiplied by 100), so that a normalised composite of 0.05 is reported as a score of 5. All QA scores stated and plotted in Section 4 follow this 0–100 convention.

The overall QA score *Q* is computed using a weighted sum of individual metrics, as defined in Equation (1):

(1) Q=0.4S+0.35D+0.25E.

The weights were tuned iteratively against a pilot set of frames that two clinicians had independently labelled as “good”, “acceptable”, or “unusable”. Starting from equal weights, the coefficients were adjusted in small steps until the rank ordering induced by *Q* broadly agreed with the clinician categorisation, i.e., frames graded “good” consistently received higher *Q* than frames graded “unusable” within the same sweep. The resulting values (placing slightly higher importance on sharp edge delineation than on grey-level dispersion) are consistent with prior phantom-based ultrasound IQA studies that identify intensity spread and Laplacian-based sharpness as among the most discriminative cues within broader metric sets [27,28,47]; edge density contributes complementary information about lumen–wall definition. We do not claim these specific coefficients are optimal in a global sense; deployment to other anatomies or imaging presets would warrant re-tuning against site-specific clinician labels, and a more principled alternative would be to learn the weighting end-to-end against expert grades, as in recent deep-learning IQA work [49,50], once a sufficiently large annotated dataset becomes available.

Frames are then ranked in descending order of *Q* to obtain the top-*k* diagnostic images for subsequent analysis. The full rule-based QA and ranking procedure is summarised in Algorithm 1.
**Algorithm 1** Aorta-Based Quality Assessment and Frame Ranking**Input:** Multi-frame DICOM sequences (Manual, Robotic)**Output:** Top-*k* ranked frames with QA metrics (Sharpness, Grey-level dispersion, Edge Density)  1:Load sequences $\left\{I_{t}^{\left(M\right)}\right\},\left\{I_{t}^{\left(R\right)}\right\}$  2:**for** each frame $I_{t}$ in both sets **do**  3:     Preprocess: convert to grayscale, crop borders, apply CLAHE and Gaussian blur  4:     Segment: $B_{t}\leftarrow$ Otsu(*I_t_*); apply morphological closing  5:     Extract contours and filter by anatomical criteria and elliptical fit  6:     Fit ellipse $E_{t}$ to best contour; expand by 20% to define region $R_{t}$  7:     Compute metrics:  8:         $S_{t}\leftarrow$ LaplacianVar(*R_t_*)  9:         $D_{t}\leftarrow$ StdDev(*R_t_*) {grey-level dispersion}10:         $E_{t}\leftarrow \frac{\left|CANNYEDGES(R_{t})\right|}{\mathrm{area}(R_{t})}$11:     Store $\left(t,S_{t},D_{t},E_{t}\right)$12:**end for**13:Normalize $S_{t}$, $D_{t}$, and $E_{t}$ to [0,1] across all frames (manual and robotic pooled)14:$Q_{t}\leftarrow 100\;\left(0.4\;S_{t}+0.35\;D_{t}+0.25\;E_{t}\right)$ {reported on 0–100 scale}15:Rank frames by $Q_{t}$ (descending)16:**return** Top-*k* frames and QA metrics

#### 3.2.3. Top-Quality Ultrasound Image Selection

In robotic ultrasound, continuous sweep acquisitions produce large frame volumes, increasing the burden on clinicians to review and select diagnostically relevant images. Building on the composite QA metric defined in Section 3.2.2, frames are ranked in descending order of *Q* and the top-*k* diagnostic images are retained for subsequent analysis. Frames are then carried forward to the downstream modules only when a valid aortic region is detected, as described in Section 3.2, ensuring that artefact-heavy frames or frames without reliable aorta localisation do not influence later outputs. The resulting top-ranked frames form the input for the automated abdominal aortic diameter measurement described in Section 3.2.4.

#### 3.2.4. Automated Abdominal Aortic Diameter Measurement

We designed a fully rule-based algorithm to estimate the infrarenal abdominal aortic diameter from individual B-mode frames acquired by the robotic system. The algorithm follows a six-stage pipeline applied to each frame: cropping, enhancement, multi-branch candidate detection, refinement, unified scoring, and millimetre conversion. The cropping and enhancement stages reuse the pre-processing and automatic aorta localisation described in Section 3.2, providing a consistent region of interest around the infrarenal aorta for subsequent analysis. This cropping restricts each frame to the aortic region of interest and removes both surrounding tissue and the peripheral on-screen annotations of the ultrasound display (the depth scale bar, subject identifiers, and acquisition text), which would otherwise introduce spurious contours and distract the automatic lumen detection from the image content around the aorta. Because the physical diameter is then derived from the DICOM pixel spacing recorded in the frame metadata (Equation (2)) rather than from any on-screen scale, the measurement remains fully metric and independent of the displayed field of view, and imaging depth is not a required reference for transverse-diameter estimation. The example panels used for diameter validation in Section 4.6 are therefore the genuine cropped algorithm inputs and outputs, which no longer contain a depth scale bar, although short fragments of the display’s on-screen text can remain at the edge of a panel; these fall outside the aortic region of interest and are not used by the algorithm.

Candidate lumen regions are then generated through a multi-branch detection pipeline combining contour analysis, circular detection, and cavity extraction. First, external image contours are fitted with ellipses, from which only geometrically plausible shapes located in the lower-middle region of the image are retained. Second, circular structures are searched using a Hough-based detector with radius bounds that scale with the image dimensions, allowing the method to capture lumens of varying apparent size. Third, a distance-transform representation is used to identify the darkest approximately circular cavity, providing robustness when edges are weak or partially missing. All candidates from these branches are then refined by evaluating lumen-wall contrast through an intensity comparison between each ellipse and its surrounding ring, discarding regions without a clear contrast boundary. The remaining candidates are ranked using the composite score ranking module that combines boundary contrast, the proportion of dark pixels, geometric plausibility, and simple positional priors, with penalties for irregular shapes or abnormal texture patterns. The top-scoring ellipse is selected as the aortic lumen, and its minor axis (short axis) is converted into a physical diameter using the DICOM pixel spacings and the ellipse orientation angle θ:

(2) $$d_{\mathrm{mm}}=\min\left(MA,\;ma\right)\;\sqrt{{\left(s_{x}\sin\theta \right)}^{2}+{\left(s_{y}\cos\theta \right)}^{2}}+2\;\mathrm{ADD}\text{\_}\mathrm{MM}$$

Here, MA and ma denote the lengths (in pixels) of the major and minor axes of the fitted ellipse, respectively, $s_{x}$ and $s_{y}$ are the DICOM pixel spacings in the horizontal and vertical directions (mm/pixel), and θ is the orientation angle of the major axis relative to the image *x*-axis. Because the reported diameter is taken along the ellipse minor axis, which is oriented at θ+90° relative to the major axis, the pixel-to-millimetre conversion is evaluated along this minor-axis direction, giving the factor $\sqrt{{\left(s_{x}\sin\theta \right)}^{2}+{\left(s_{y}\cos\theta \right)}^{2}}$ (equivalently, replacing θ by θ+90° in the major-axis projection). In this study the scan-converted B-mode DICOM frames have isotropic pixel spacing ($s_{x}=s_{y}=s$), so this factor reduces to *s* and the estimated diameter is independent of θ; the orientation-dependent form is retained for the general anisotropic case. The term ADD_MM is an optional additive margin in millimetres (e.g., to account for vessel wall thickness), which was set to zero in this feasibility study.

This method does not rely on machine learning or deep learning, thus avoiding the need for annotated training data and remaining computationally lightweight for real-time or near-real-time use on standard hardware.

### 3.3. Transfer-Learned U-Net Segmentation Baseline

As a learning-based reference for the rule-based pipeline of Section 3.2, we trained a transfer-learned U-Net [58] to segment the abdominal aorta on robotic-ultrasound frames. This baseline is used only for offline comparison and does not contribute to the screening outputs reported in this study; it is included so that the rule-based choice can be benchmarked against a standard learning-based alternative on the same acquisition data.

Training followed a three-stage simulated-to-real strategy to mitigate the scarcity of labelled clinical data. Stage 1 used the public ultrasound dataset of Vitale et al. [59]: of the 926 simulated frames (ray-cast from CT), 334 were retained after filtering for visible vasculature, with binary vessel masks split 80/20 train/validation and the training portion tripled via standard augmentation (rotation, shift, shear, zoom, flip). Stage 2 fine-tuned on 10 expert-annotated real frames using 5-fold cross-validation across three optimisers (Adam [60], RMSprop [61], SGD [62]); encoder weights were transferred from the Stage-1 model and the decoder was re-initialised to learn real-ultrasound reconstruction patterns. Stage 3 retrained the best Stage-2 configuration on an expanded set of 48 expert-annotated real frames (38 train/10 test) with early stopping. The network uses a standard four-block encoder–decoder with batch normalisation, LeakyReLU activations, dropout, and skip connections, trained with a combined binary cross-entropy + Dice + focal loss [63,64] to address the small-vessel class imbalance and ambiguous boundary regions. Segmentation quality was assessed using the Dice coefficient (primary metric), with IoU, precision, recall, and F1 as secondary metrics.

For mask-derived diameter estimation, the maximum-inscribed circle inside a vessel mask was obtained via a Euclidean distance transform, and a depth-correction factor (fitted by linear regression) converted the radius to physical units across volunteers with different imaging depths. In a deployed learning pipeline this mask would be the U-Net’s own prediction; in the offline validation reported here, however, the same extraction is applied to the clinician ground-truth masks as an oracle (perfect-segmentation) upper bound, for the data-availability reasons explained in Section 4.

### 3.4. Volunteer Testing Experimental Protocol

This study was approved by the King’s College London local ethics committee (study title: Investigating Robotic Abdominal Ultrasound Imaging, reference: HR-22/23-5412), and all volunteers provided written informed consent before participation. Healthy adults (≥18 years) were recruited through internal advertisements. To evaluate performance across a wide range of body types, recruitment explicitly sought variation in sex/gender, age, body habitus (BMI), and skin colour. Exclusion criteria included pregnancy, recent abdominal surgery, implanted abdominal devices, intolerance to ultrasound gel or probe pressure, and any condition that a clinician judged unsafe for scanning.

The hardware setup is shown in Figure 2a and described in Section 3.1.1. To ensure participant safety, the study was conducted with strict protective measures. Maximum axial force limits were enforced both in software, through a force-control algorithm, and in hardware, through a mechanical clutch. A physical emergency-stop button was held by the volunteer, allowing immediate shutdown of all system power if pressed. In addition, a trained operator supervised every session and could take manual control at any time.

**Figure 3:**
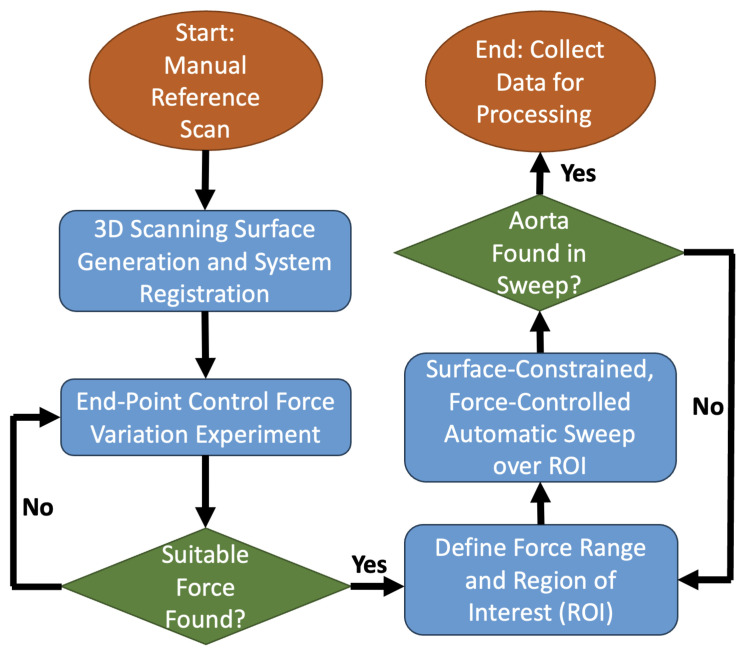
Structured workflow of volunteer experiments in robotic abdominal aortic ultrasound.

Each volunteer session followed a structured workflow (Figure 3) with four main steps. The overall aim of this four-step protocol was to verify two key capabilities of the system: (1) reliable aorta visualisation under endpoint control and (2) completion of automatic surface-constrained sweeps under force control and target tracking.

**Figure 4:**
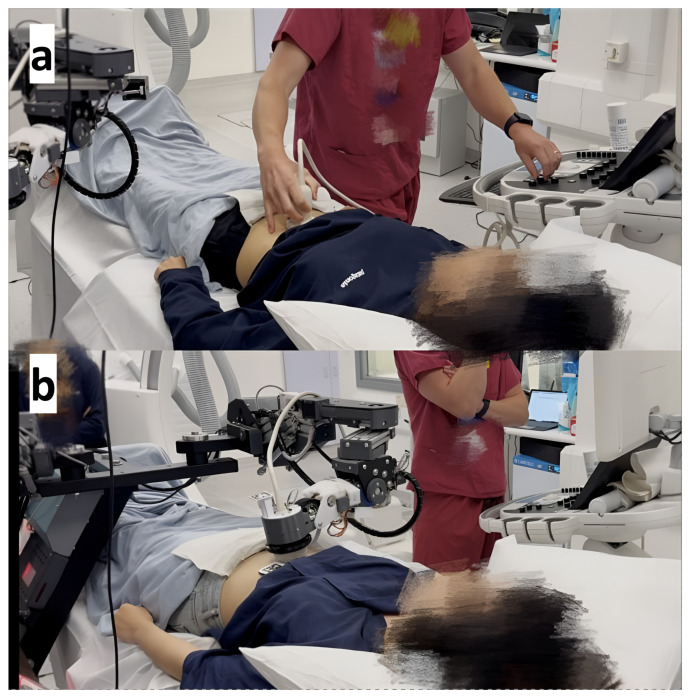
Stages of volunteer abdominal aorta ultrasound scanning protocol. (**a**) Manual reference scan performed by a trained clinician to localise the abdominal aorta and record probe force. (**b**) Robotic scan performed on the same volunteer using the autonomous system with integrated force control and patient-to-robot registration, under operator supervision.

All volunteer scans were acquired by a single NHS-registered clinical vascular scientist to ensure consistency. Image quality grades and reference diameter measurements were then reviewed by two NHS-registered clinical vascular scientists (including the scanning operator), both specialising in abdominal aortic ultrasound; these are hereafter referred to collectively as the “clinicians”. To establish a reference for comparison, the clinicians first performed a brief manual scan. This scan localised the abdominal aorta, identified the clearest diagnostic frame, and recorded the axial probe force using the integrated force sensor (Figure 4a). While this manual scan provided a reference image and force profile, the robotic workflow itself does not depend on prior human input and is designed to operate autonomously in clinical use. AprilTag markers were then attached to the volunteer’s abdomen and to the robot, and an RGB-D surface capture established the patient-to-robot registration. These markers also support real-time motion tracking during scanning.

The robot subsequently performed three supervised experimental tasks (Figure 4b). Firstly, in the Force Variation Experiment, the probe was positioned over the aortic region under direct end-point control. Images were then collected at multiple force levels bracketing the manual reference. These data allowed determination of a participant-specific target force band that balanced image visibility, stable contact, and comfort.

Secondly, the operator defined the region of interest (ROI) on the reconstructed 3D surface and confirmed the target force band to be used for autonomous execution. Thirdly, the robot carried out a surface-constrained automatic sweep over the ROI, employing hybrid position–force control to maintain both the desired force band and local surface normal alignment. If the aorta was not clearly visualised, the sweep was repeated with adjusted parameters until satisfactory diagnostic images were obtained. We recorded the success rate, time to reacquire, and any operator interventions.

Sessions concluded with the robot returned to a neutral pose, markers removed, gel cleaned, and participants thanked and released. These objectives were tested across all volunteers, with quantitative logging of original ultrasound images, sweep duration, force readings, and pose trajectories. Image quality was assessed by two registered clinicians and cross-validated against automated algorithms.

The decision to use a force-driven scanning protocol, rather than image-driven control, was guided by both patient safety and system scalability. Abdominal ultrasound requires significantly variable contact force depending on patient body habitus, particularly BMI. In some cases, optimal image quality may require probe forces exceeding 30 N, which, when applied by a robot, can cause discomfort or distress. By tailoring the force band for each participant, we ensured stable imaging while maintaining patient comfort and safety. Additionally, image-driven control necessitates real-time access to ultrasound image streams, which is difficult to standardise across diverse ultrasound systems worldwide. In contrast, force control is implemented entirely within our integrated hardware-software platform, supporting broader deployment without dependency on external imaging hardware.

### 3.5. Volunteer Questionnaire Design

A post-scan questionnaire was administered to all volunteers, designed under ethical approval (King’s College London Research Ethics Committee Ref: HR-22/23-5412). The questionnaire consisted of 17 questions divided into three categories (ethical procedure and communication, perception of the robotic system, and comfort and experience during the scan), matching the structure of the results presented in Section 4.8.

## 4. Results and Discussion

### 4.1. Volunteer Demographics and System Reliability

A total of ten healthy volunteers were recruited for this study (Table 1), comprising seven males and three females. This distribution reflects the clinical prevalence of AAA, which are significantly more frequent in men than women, and thus ensures a representative validation setting. Participants were drawn from the 20–60 age range, although most were in the 20–30 group due to the characteristics of our ethically approved volunteer pool. BMI values in our volunteer groups spanned from 18.6 to 33, thereby covering both normal-weight and obese body habitus according to World Health Organization (WHO) criteria. Specifically, BMI <18.5 is underweight, 18.5≤BMI<25.0 is normal weight, 25.0≤BMI<30.0 is overweight, and BMI≥30.0 is obese. This diversity is important since abdominal fat content can strongly affect ultrasound penetration and image quality. Cross-variation was also considered: both males and females were represented across higher and lower BMI values, avoiding bias such as only recruiting slim females. Skin tone diversity was included, with White, Brown, and Asian participants. This ensured that the system was tested under different acoustic and tissue attenuation conditions. Together, this cohort provided a heterogeneous yet clinically relevant test population to assess the robustness of the robotic imaging workflow across sex, age, body composition, and demographic diversity.

Scanning time varied substantially between manual and robotic acquisitions. For the experienced clinicians, manual localisation of the abdominal aorta typically required only 30 s in slim volunteers. In contrast, it could extend up to 3 min in participants with higher BMI or increased bowel gas, where acoustic windows were more challenging. The robotic workflow’s final automatic sweep was comparable in efficiency, taking about 30 s in slim cases and approximately 1 min in high-BMI volunteers. However, the robotic procedure includes several preparatory steps: system calibration, the force variation test, and region-of-interest (ROI) and force-band selection. These are essential to ensure safe probe contact and reliable image quality. When these steps are included, the full robotic session required between 15 and 30 min, depending on participant characteristics and scanning conditions.

To evaluate robustness, we monitored failures and interruptions during all volunteer and phantom sessions. Across the ten volunteer sessions, conducted over four separate days within a two-month period, the robot accumulated approximately 8 h of operation in volunteer testing and over 20 h in phantom experiments. During this time, neither the hardware (mechanical clutch, force sensors, motor system) nor the software (registration, force control, path planning) experienced any breakdowns.

A few non-critical interruptions were observed. In some cases, the selected scanning area exceeded the robot’s physical workspace, prompting the system to pause; this was easily resolved by homing the robot and slightly repositioning the volunteer. On rare occasions, the force sensor signal would briefly pause after more than one hour of continuous operation. The system automatically detected this condition, reconnected the sensor within one second, and safely waited for the sensor to repower before resuming operation. Importantly, the emergency stop button held by each volunteer was never pressed, indicating that the robot did not behave unexpectedly or cause discomfort.

In all ten volunteers, the robotic acquisitions yielded clinically usable abdominal aorta images, as confirmed by independent clinician review, indicating that diagnostic information could be obtained in every case. Detailed analyses of image quality, force–image relationships, and automated aorta measurement are presented in the following sections.

### 4.2. Force-Control Robustness Across BMI

**Figure 5:**
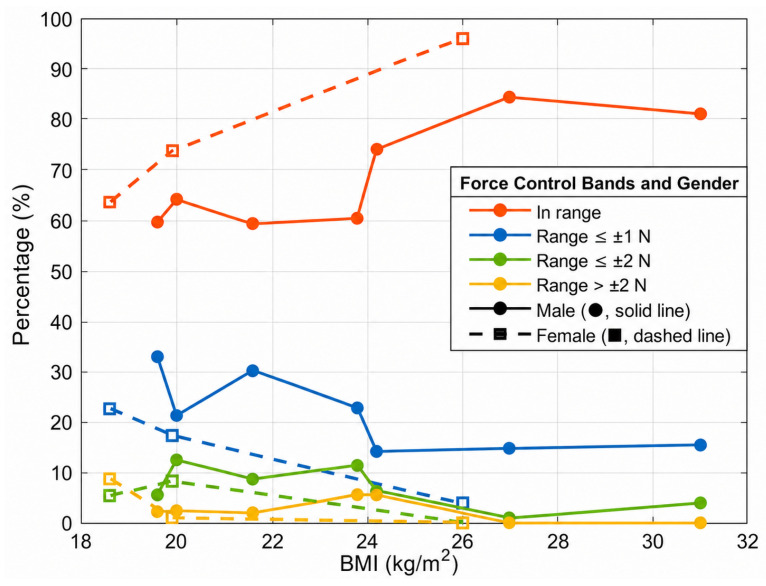
Force control performance across BMI ranges. The percentage of time spent within different force bands is plotted for male (solid lines) and female (dashed lines) volunteers.

During each automatic sweep, every force sample was logged and classified into four tolerance bands: in-range, within ±1N, within ±2N, and >±2N (Figure 5). Across all ten sessions, the controller maintained stable contact, holding an average 71.6% of samples within the target band (range 59.3–96.0%). Most deviations were minor: 19.4% falling within ±1N, 6.3% within ±2N, and only 2.7% exceeded ±2N, indicating smooth and safe force regulation.

**Figure 6:**
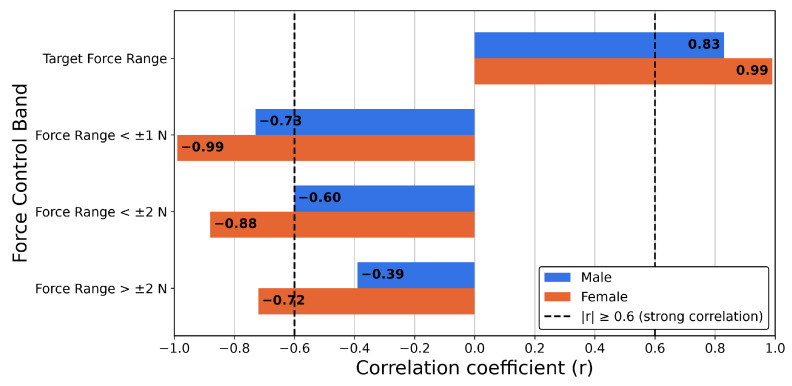
Correlation between BMI and force control performance by gender. Bars show Pearson correlation coefficients (*r*) for male and female volunteers across four force-control bands. Dashed lines indicate the threshold for strong correlation (|r|≥0.6).

A clear dependence on BMI was observed. As shown in Figure 6, the in-range proportion increased with BMI, with a significant positive correlation (Pearson r=0.68, p=0.03). When analysed by gender, a similar positive trend was seen in both subgroups (males: r=0.83, p=0.02; females: r≈0.99, n=3), although the female correlation should be interpreted with caution given the very small sample size. Negative correlations were observed for out-of-range bands (e.g., r≈−0.7 for ±1N), indicating that higher BMI was associated with fewer large force deviations. These results suggest that participants with greater body mass generally enabled steadier and more stable force regulation under robotic control.

Beyond the overall BMI trend, exploratory subgroup analysis suggested that female volunteers tended to achieve higher in-range fractions than males with comparable BMI (mean 77.7% vs. 68.9%; n=3 females, n=7 males), although this difference was not statistically significant (Welch’s *t*-test p=0.47). This pattern is consistent with the hypothesis that, at similar BMI values, females typically have a higher proportion of subcutaneous fat and softer abdominal tissue, potentially providing a more compliant probe–tissue interface and smoother contact. However, given the small number of female participants, this should be viewed as a qualitative trend rather than a definitive statistical effect. Together, these observations indicate that both BMI and underlying body composition may influence robotic force-control stability, with higher adiposity plausibly favouring smoother probe–tissue interaction.

We stress that all correlation and significance values reported in this and the following subsections are exploratory and should be interpreted as hypothesis-generating rather than confirmatory. With only ten volunteers, and in particular only three female participants, individual coefficients are estimated from very few points, are highly sensitive to single observations, and are not corrected for the multiple comparisons made across force bands and BMI subgroups. The near-perfect female correlation (r≈0.99, n=3) is a striking illustration of this: a coefficient computed from three points is essentially unconstrained and carries no reliable evidential weight. We therefore avoid drawing firm quantitative conclusions from these fits and instead report them only to indicate qualitative trends; establishing statistically robust BMI-, sex-, and body-composition effects will require the larger, prospectively powered cohort discussed in Section 5.

### 4.3. Comparison of Manual and Robotic Image Quality

In this section, we compare the composite image quality of manual and robotic scans using the frame-level QA metric defined in Section 3.2.2 and the frame-selection procedure described in Section 3.2.3. Based on discussions with the clinicians and direct observation of abdominal aorta examinations in the clinic, we noted that human operators often apply relatively high probe forces, particularly in higher-BMI patients, in order to obtain an optimal view of the aorta. To quantify this, we mounted a force sensor on the ultrasound probe and monitored contact force during all scans. Peak forces during ten manual examinations ranged from 5 to 55 N (mean ≈23 N, median 15 N), with several cases requiring forces above 40–50 N. In contrast, for safety and patient comfort, the robotic controller was deliberately constrained to contact forces below 15 N and therefore could not reproduce the highest manual forces. Within this constraint, we performed experimental robotic sweeps while two clinicians observed the live images and marked frames that provided a clinically usable view of the abdominal aorta. Both reviewing clinicians agreed that, despite the conservative force limit, the robot was able to acquire images that were clinically acceptable for AAA screening.

**Figure 7:**
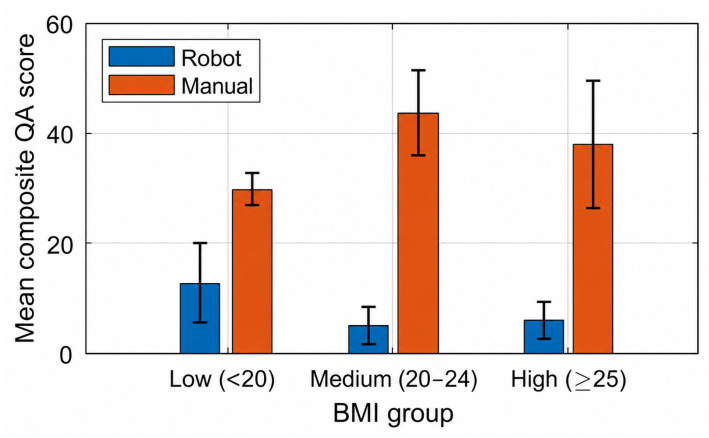
Composite QA scores for robot and manual scans across BMI groups. Bars show mean composite QA score for frames/images judged clinically usable; blue bars correspond to robot sweeps and orange bars to manual scans. Error bars indicate ±1 standard deviation.

We then related these clinical judgements to the quantitative image-quality metric, as summarised in Figure 7. The ten clinician-selected manual reference images achieved consistently high composite QA scores (36.4±9.1), with all values in a relatively narrow band of approximately 25–50 and with similarly high means (around 30–44) across all BMI groups. In comparison, the 149 frames acquired during the robotic sweeps had lower and more variable scores (8.5±6.3), with most values below 20 and best performance in low-BMI participants (mean ≈12.8) and lower means of ≈5–6 in medium- and high-BMI groups.

Within the pooled robotic set, the frames the clinicians judged diagnostically usable occupied the upper part of the robotic distribution (lowest accepted score ≈5, against a robotic mean of 8.5±6.3), and these frames were consistently associated with contact forces of at least about 5 N. Because the composite score rises with contact force before plateauing in most volunteers (Section 4.4), we use Q≳5 as an operating criterion for selecting the contact-force window, not as a per-frame acceptance test. Absolute scores are bounded by the acoustic quality attainable in a given subject, so a clinically usable frame may score well below 5 (Section 4.5); within a single acquisition the score serves only to rank frames (Section 3.2.3). In the remainder of this section we therefore use the force range observed for the accepted frames (typically around 8–12 N, with a lower bound near 5 N) as the basis for the subsequent force-image-quality analysis.

We emphasise that Figure 7 is not intended as a like-for-like “manual versus robotic” superiority test, and the lower robotic mean should not be read as evidence that robotic imaging is inherently inferior. The system does not aim to reproduce the clinician’s contact force or probe pose; under its own registration, surface-constrained path planning and force control, it reaches the views it judges best, so the relevant question for screening is the quality of these outcomes rather than replication of the manual process. The two distributions are also deliberately asymmetric in two respects. First, the manual set consists of a single clinician-selected best diagnostic frame per volunteer (ten frames in total), i.e., the upper extreme of the manual quality distribution, whereas the robotic set comprises all 149 frames spanning entire sweeps, including transitional and off-target frames that a clinician would normally discard; comparing a curated best frame against a full continuous acquisition necessarily favours the former. Second, the two acquisitions operate in different force regimes: the manual examinations used peak forces up to 55 N (mean ≈23 N), whereas the robot was deliberately capped below 15 N for safety and comfort, which limits acoustic penetration in deeper or higher-BMI anatomy and therefore caps the achievable composite score. The clinically relevant observation is not the difference in means but that, even under a conservative force limit and without best-frame curation, the robotic sweeps still yielded frames that both clinicians judged diagnostically acceptable and that ranked at the top of their own sweeps. Integrating a real-time frame-selection loop (Section 5) is a natural next step, allowing the robot to retain only its highest-quality diagnostic frames during acquisition.

### 4.4. Effect of Contact Force on Image Quality

Abdominal aorta imaging is highly force-dependent because the vessel lies deep in the abdomen, especially in higher-BMI patients. Having established that the frames clinicians accepted as diagnostically usable occur at forces of roughly 5 N and above (most commonly 8–12 N), we further investigated how force and image quality interact across different BMI groups.

**Figure 8:**
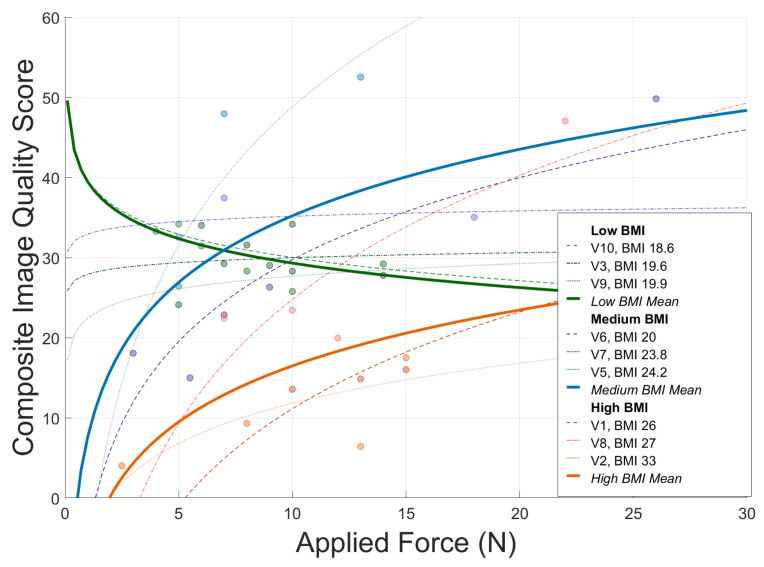
Composite metric score versus applied force for all volunteers. Everything in the plot is colour-coded by BMI group: low (green), medium (blue), and high (orange). The coloured dots are the individual acquired frames, each plotted at the contact force at which it was recorded against its composite QA score; these are the observations to which the curves are fitted. The thick solid curves are the group-mean logarithmic fits, and each thinner dashed or dotted curve is the logarithmic fit for one individual volunteer (identified by ID and BMI in the legend).

A force sensor was mounted on the ultrasound probe, and an endpoint control strategy was used to acquire images under different applied forces for each volunteer. In these experiments, the nominal robotic force range was set to approximately 8–12 N, with small excursions outside this band during motion. The analysis used data from 9 of the 10 volunteers; one scan was acquired with a different ultrasound imaging preset, resulting in contrast characteristics that were not comparable to the remaining dataset, and was therefore excluded from this sub-analysis. Figure 8 presents the relationship between applied force and composite image quality scores for volunteers grouped by BMI. Solid lines represent the averaged trends for low-, medium-, and high-BMI groups, while thinner dashed/dotted lines correspond to individual volunteers within each group. Logarithmic fitting was applied to illustrate the plateauing trend between increasing force and image quality.

As shown in Figure 8, volunteers with a low BMI (green) achieved high composite scores (30–40) with relatively small applied forces, and further increases in force had minimal effect (r=−0.33, p=0.26). In contrast, the medium-BMI group (blue) exhibited a clear positive correlation between force and image quality, which plateaued at higher force levels (r=0.69, p=0.013). The high-BMI group (orange) followed a similar trend but consistently achieved lower composite scores at comparable force levels (r=0.44, p=0.13). Across all BMI groups, the group-mean curves both exceed the Q≳5 operating criterion of Section 4.3 and have entered their plateau by the 8–12 N range, so additional force beyond this band buys little further image quality while increasing the load on the abdomen. This identifies 8–12 N as a practical operating window for the robot: as long as probe position and orientation are appropriate, maintaining contact forces within this range gives confidence that clinically usable aortic images can be obtained for AAA screening.

This criterion presumes that pressing harder improves image quality, which holds for the low- and medium-BMI volunteers: for these participants Figure 8 identifies a force window that comfortably exceeds the operating level, and the clinicians accepted the frames acquired within it. The premise is weakest exactly where screening is hardest. For the high-BMI group the force–quality association is positive but not significant (r=0.44, p=0.13) and the scores stay low throughout; in the BMI-33 participant the aorta lies deep enough that acoustic penetration, rather than coupling, is limiting, so force offers little further leverage and cannot be raised indefinitely without compromising comfort. Section 4.5 therefore examines this participant directly.

Notably, our single high-BMI participant (Volunteer 2; BMI =33 and aged 50–60, i.e., the individual closest to the typical AAA screening profile) still yielded aortic images that both clinicians rated clinically usable under the 15 N cap, with an automated diameter within 0.1 mm of the clinician measurement (Table 2), albeit at systematically lower composite quality scores than lower-BMI subjects (Figure 8). The system therefore did acquire a diagnostic view in this case, and the limitation is one of generalisation rather than outright failure: a single high-BMI participant does not let us claim that the 15 N cap will suffice for every obese patient. This is also consistent with the system’s intended use as a first-line screening tool for the general population, automating the routine examinations of frontline sonographers rather than replacing expert assessment of the most difficult cases, which in routine practice are triaged to more experienced clinicians using specialised techniques (higher contact force, alternative probes, positioning). Reduced image quality in obese abdomens is, moreover, largely an inherent acoustic-penetration limitation that affects manual scanning as well, so it reflects patient anatomy as much as the robot’s force cap. A dedicated high-BMI study, together with penetration-preserving options (curved-array probes, patient positioning, and a supervised mode permitting brief, monitored higher-force excursions), is left to future work (Section 5).

### 4.5. Agreement Between Automated QA and Clinician Grading

We next examined how the automated QA scores from Section 3.2 align with clinician-assigned visual quality grades for a challenging robotic sweep. The sweep analysed here is the autonomous acquisition on the BMI-33 participant, at the highest contact force the volunteer found comfortable. Since the force-based criterion cannot be relied upon in this subject, we ask instead, by direct frame-by-frame grading, whether diagnostically usable images are obtained at all and what scores they attain. Each frame index from the DICOM sweep was manually reviewed and labelled by the clinician using four quality categories: ‘Good’, ‘Acceptable’, ‘Unusable’ and ‘Bifurcation’. The labels were mapped to the corresponding frame indices, ensuring accurate pairing between visual labels and detection score outputs.

**Figure 9:**
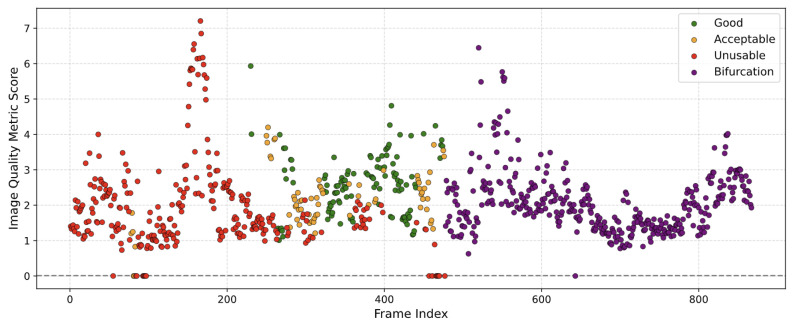
Frame-level alignment between automated QA metrics and clinician visual quality grades. Coloured markers denote clinician-assigned categories (Good, Acceptable, Unusable, and Bifurcation), allowing direct comparison with the automated scores across the full sweep. Scores are on the same 0–100 composite scale as Figure 7 and Figure 8. This is a single continuous sweep acquired on the highest-BMI participant under a comfort-limited contact force, and its scores therefore occupy only the lowest part of that scale.

Figure 9 compares the QA metric scores with the clinician-assigned grades, enabling a direct link between detection reliability and expert perception. Figure 9 uses the same metric, the same pooled min–max normalisation and the same 0–100 scale as Figure 7 and Figure 8 (Section 3.2.2); no separate scaling is applied. Its narrower range (roughly 0–7) reflects the data. The earlier figures pool the whole cohort, and their upper range is set by the low-BMI volunteers; this is a single dynamic sweep on the highest-BMI participant, in which probe motion and out-of-plane anatomy further depress the score relative to the stationary, force-controlled acquisitions of Figure 8. Frames graded ‘Good’ or ‘Acceptable’ here score only about 1–4. This is the finding of the experiment, not a contradiction of it: the Q≳5 criterion selects a contact-force window and is not applied as a frame-acceptance threshold in this figure. The distribution of labels shows that early sweep segments were predominantly unusable, followed by a mid-sweep region with clinically acceptable frames, and later segments dominated by bifurcation labels, consistent with anatomical transitions near the aortic bifurcation. Overall, frames graded as ‘Good’ (green) tended to appear in regions with higher QA scores, while low-score regions contained a larger proportion of ‘Unusable’ (red) and ‘Bifurcation’ (purple) labels. This indicates that the metric broadly reflects the clinician’s perception of image quality, but with several notable exceptions. For example, several high-score peaks just before frame 200 and after frame 500 were labelled ‘Unusable’ and “Bifurcation” by the clinician. On closer inspection, these frames exhibited good overall image quality (sufficient force and satisfactory greyscale appearance) but did not yet include the aorta within the field of view, as the probe had not reached the correct anatomical region. This explains the large apparent discrepancy and highlights an important limitation: the automated QA metric evaluates generic image quality rather than verifying that the aorta is actually present, so high scores do not always imply clinical usefulness for aorta-focused assessment.

We next quantify the diagnostic yield of the sweep. The clinician’s ‘Unusable’ grade conflates two distinct situations: frames in which the aorta is not in the imaging plane at all, and frames in which the aorta is visible but the image is too poor to assess. The first is expected at the start of a sweep, while the robot is still settling its contact force and searching for the vessel, and it says nothing about image quality; several such frames in fact score highly (the peaks near frame 200 noted above). Only the second reflects a shortcoming of our force control and imaging pipeline, and it is the quantity of interest here. We therefore restrict the denominator to the window running from the first frame graded ‘Good’, which marks the aorta entering the field of view, to the first frame labelled ‘Bifurcation’, beyond which a single ellipse no longer models the lumen; in Figure 9 this is the span from the first green marker to the first purple one. Within this window an ‘Unusable’ grade carries only its intended meaning. Of these frames, 29.85% were so graded, so just over 70% gave a usable view of the aorta. This is a clinician-only measure: it involves no QA threshold and is therefore unaffected by the compressed score range of the figure. Even at BMI 33, under autonomous motion and a contact force limited by the volunteer’s comfort, the robot spent most of the sweep acquiring frames a clinician would accept. This is a stronger result than the system was designed for. Our target is the routine screening of the general population, not the difficult abdomen, which in current practice is triaged to an experienced operator; that a fully autonomous sweep still returns a diagnostic yield of over 70% at the top of the BMI range suggests the approach degrades gracefully rather than failing there. We are nonetheless careful not to generalise from a single volunteer and a single sweep. Establishing that the system remains effective at high BMI requires the larger cohort recruited from the older and higher-BMI screening population, and the dedicated high-BMI study, both set out in Section 5. The temporal evolution of the scores also offers an indirect assessment of model robustness: smooth progression suggests dependable performance, whereas abrupt fluctuations may reflect sensitivity to artefacts.

### 4.6. Rule-Based Aortic Diameter Estimation

**Figure 10:**
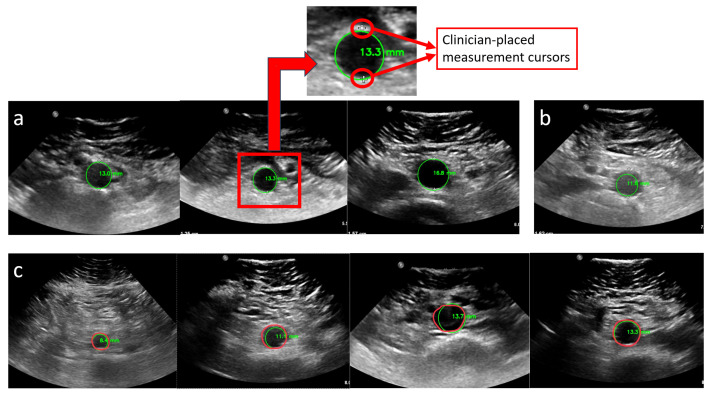
Abdominal aortic diameter measurement and validation in manual and robotic scans. (**a**) Example manual scans with clinician ground-truth measurements (white cursors) and closely matching automatic diameter estimates (green circles), with errors within 1 mm. (**b**) Example manual scan illustrating a more challenging case with a less accurate automatic estimate (green circle), but the error is still within 5 mm. (**c**) Example robotic sweep frames with clinician-drawn aortic contours (red circles) and overlapping automatic estimates (green circles). Frames are cropped to the aortic region of interest during preprocessing, which also removes the ultrasound display’s peripheral annotations (depth scale bar, subject identifiers, and acquisition text); the panels shown are thus the genuine algorithm inputs and outputs. Because the crop is anchored on the aorta rather than on the display, short fragments of the original on-screen text remain visible at the lower edge of some panels; these lie outside the aortic region of interest and are not used by the algorithm. Because the diameter is computed from the DICOM pixel spacing (Equation (2)) rather than a displayed scale, absolute size is preserved even though no depth scale bar is present.

To evaluate the proposed rule-based diameter extraction algorithm, we analysed ten validation images (one per volunteer). For each volunteer, the clinician first performed a manual scan and measured the infrarenal aortic diameter directly on the ultrasound machine, following routine clinical practice. These clinician-derived measurements were treated as ground truth. Using the rule-based diameter extraction pipeline described in Section 3.2.4, the algorithm then automatically estimated the diameter on the corresponding images. The resulting automated diameters were compared with the clinician measurements recorded on the ultrasound device, as summarised in Table 2, where the Calculated Diameter refers to the algorithm output and the Actual Diameter to the clinician measurement. Figure 10a shows examples of manually acquired validation frames: the white measurement cursors indicate the clinician’s on-machine diameter measurements (ground truth), and the green circles show the automatically estimated aortic lumen and diameter. Figure 10b illustrates a more challenging manual case (Volunteer 5), corresponding to the largest observed error. Figure 10c then shows representative frames from robotic automated sweeps, where on-machine measurements were not taken in real time; instead, the clinician retrospectively outlined the aortic boundary (red circles), which overlaps closely with the green automatic circles.

In line with studies reporting that trained operators can achieve inter- and intra-observer variability of approximately 2–3 mm in abdominal aortic diameter measurements [65,66,67], a difference within 3 mm between automated and clinician measurements was adopted as an acceptable agreement threshold. Under this 3 mm criterion, the algorithm produced diameter estimates within 3 mm for 9/10 cases. The remaining case (Volunteer 5, difference 4.6 mm) represents a challenging image in which the infrarenal aorta is only weakly visualised. This can occur, for example, when imaging settings such as gain are not fully optimised, reducing blood–wall contrast and causing the lumen to appear overly bright (Figure 10b). Although the rule-based pipeline slightly underestimated the diameter, the error remained within the 5 mm variability commonly reported for ultrasound AAA measurements.

Across all cases, the mean signed difference (automated minus clinician) was −0.55 mm (Table 2), yielding a small negative mean offset. Signed errors were evenly split between over- and underestimation (five each), spanning from −4.6 to +1.3 mm, with the most negative deviation occurring for Volunteer 5. Excluding Volunteer 5, the mean signed difference reduced to −0.10 mm, suggesting the apparent underestimation is largely driven by this single challenging case.

One likely contributor to any residual offset is a convention mismatch in calliper placement. In vascular ultrasound, common conventions include inner-to-inner (ITI), outer-to-outer (OTO), and leading-edge to leading-edge (LELE) [68,69]. Here, the clinician followed the LELE convention, which correlates well with CT-based external diameter measurements [70], whereas the automated pipeline measures the lumen’s inner boundary, corresponding more closely to ITI. This difference in boundary definition can reasonably account for an approximately ∼1 mm systematic discrepancy. Notably, the NHS Abdominal Aortic Aneurysm Screening Programme recommends an inner-to-inner convention for screening [71], so the observed offset is better interpreted as a measurement-convention difference rather than inferior algorithm performance.

Qualitative inspection of Figure 10 and the collected dataset indicates that successful detections tend to occur when (1) a clear intensity contrast exists between the aortic lumen and surrounding tissue, and (2) the aortic cross-section appears within the typical middle region of the ultrasound frame. Combining numerical and visual assessment in both manual and robotic scans, we conclude that for standard abdominal ultrasound images with moderate contrast and adequate depiction of the aortic cross-section, this non-AI, non-manual diameter extraction method can reliably provide a one-shot usable estimate, supporting its potential as an automated pre-screening or auto-labelling module in robotic or clinician-assisted ultrasound workflows.

A key limitation of the current implementation is that the lumen-detection and diameter-measurement stages assume an approximately circular or elliptical cross-section, which is appropriate for the healthy aortas examined in this cohort but is not guaranteed in pathological anatomy. In genuine AAA cases, the lumen may be markedly non-circular due to asymmetric dilation, focal saccular bulges, or partial occlusion by intraluminal thrombus, and the outer wall may be deformed by adjacent structures [23,24,25]. Under such conditions, the ellipse-fit short-axis used here would not necessarily correspond to the clinically relevant maximum transverse diameter, and the geometric model would need to be adapted for such shapes, for example by fitting to the outer wall rather than the patent lumen. Robust handling of these cases will likely require relaxing the geometric prior (for example, by using thrombus-aware segmentation or contour-following methods on the outer wall) and validation in a pathological cohort; this is discussed further in Section 5.

### 4.7. U-Net Segmentation Baseline Results

For completeness, we report the offline performance of the transfer-learned U-Net baseline described in Section 3.3 and compare its downstream diameter output with the proposed rule-based pipeline.

After Stage 1 simulated-data training, the model reached a mean Dice of 0.87 on the refined set of 334 simulated frames with clearly visible vessel boundaries (IoU 0.77, specificity 0.998). Stage 2 fine-tuning on the initial 10 real frames identified Adam (learning rate $10^{-4}$; mean cross-validation Dice 0.71) as the strongest optimiser, outperforming RMSprop (0.41) and SGD (0.62). Stage 3 retraining on the expanded 48-frame real dataset reached a mean Dice of 0.92±0.03 and IoU of 0.86±0.06 on the 10 unseen test frames (Table 3), with the lowest individual test score at Dice 0.84. The training Dice plateaued at ∼0.87 after approximately 100 epochs without overfitting. These results are encouraging given the limited real-data volume, but larger-scale evaluation is required to confirm generalisation.

Two points bound the interpretation of this Dice value. On the one hand, the model was first pre-trained on an independent public dataset (Stage 1), and the 10 Stage-2 fine-tuning frames were manual scans, whereas the Stage-3 training and test frames were robotic scans acquired with different probe force and pose; the fine-tuning frames are therefore distinct in both content and acquisition from the evaluation frames. On the other hand, all of these real frames come from the same ten volunteers, so the Stage-3 test frames share subjects with the training frames, and on so small a dataset we cannot claim strict subject-level independence. The Dice of 0.92 should therefore be read as an encouraging within-sample estimate rather than a demonstration of cross-subject generalisation. As the U-Net serves only as an offline baseline that does not contribute to the screening results, this does not affect our conclusions, and a subject-held-out evaluation on a larger cohort is left to future work.

To assess whether segmentation-derived measurements could in principle match clinician on-machine values, we applied the maximum-inscribed-circle diameter algorithm to clinician-provided ground-truth (GT) masks (n=92 frames across the ten volunteers). We stress that Table 4 is computed from the GT masks, *not* from the U-Net’s own predicted masks. It therefore does not measure the end-to-end accuracy of the learning-based pipeline; rather, it characterises the diameter-extraction step in isolation and represents an optimistic upper bound (an “oracle” segmentation) on what a learning route could achieve if its masks were perfect. Using the GT masks here is also a practical necessity: because the U-Net was trained on 38 of the 48 real frames, leakage-free predicted masks are available only for the 10 held-out test frames, which do not cover all ten subjects with the 3–15 frames per volunteer required for this per-subject validation, whereas GT masks exist for all annotated frames. A true end-to-end U-Net diameter would additionally inherit the segmentation error implied by the Stage-3 test Dice of 0.92, and would be expected to be somewhat larger; quantifying it on the U-Net’s predicted masks requires a held-out cohort large enough to support subject-level evaluation and is left to future work (Section 5). The per-subject results (Table 4) gave a mean absolute error of 1.4 mm and mean signed difference of +0.12 mm relative to clinician on-machine measurements, satisfying the 3 mm acceptability criterion [65,66,67] in 9/10 subjects and the 5 mm criterion in all subjects. The largest discrepancy (+4.0 mm for Volunteer 4) is partly attributable to the small number of annotated frames (n=3) for that subject; differences in acquisition depth and session calibration also contribute to the residual error.

Both routes therefore satisfy the 3 mm criterion in 9/10 cases and the 5 mm criterion in 10/10 cases: the rule-based pipeline (Table 2, signed difference −0.55 mm, range −4.6 to +1.3 mm) and the mask-derived route (MAE 1.4 mm, signed difference +0.12 mm) achieve comparable downstream error magnitudes. These two tables are not sampled identically, and a strictly frame-matched comparison is neither feasible nor the appropriate target. Even for a human operator, selecting the “best” diagnostic view is subjective and varies slightly between repetitions, so an exact frame-for-frame correspondence between manual and automated acquisitions cannot be defined. Our system, moreover, is not designed to reproduce the clinician’s exact probe position and pose; under patient-to-robot registration, surface-constrained path planning, and force control, it independently reaches the view that it judges best. What matters for screening is therefore outcome equivalence rather than process identity: whether the acquired frame adequately localises the aorta and yields a diameter that agrees with the clinician’s within the accepted tolerance. Accordingly, each route is evaluated on its own representative frames (a single clinician-selected diagnostic frame per volunteer for the rule-based pipeline in Table 2, and a per-subject mean over 3–15 annotated frames for the GT-mask route in Table 4) against the same clinician on-machine ground truth, and both fall within the same 3/5 mm acceptability envelope, which is the comparison of interest here. Given this similar measurement performance, we adopt the rule-based pipeline as the primary analysis route in this feasibility study because it is lightweight, transparent, and does not require annotated training data. We did not measure the U-Net’s end-to-end diameter directly, so this comparison should be read with caution; nonetheless, since the GT-mask route is a perfect-segmentation (oracle) upper bound that already only matches the rule-based error (1.4 versus 1.45 mm mean absolute error), a U-Net operating on its own imperfect masks (Stage-3 Dice 0.92) would be unlikely to surpass the rule-based pipeline and could plausibly be somewhat less accurate. This is consistent with, rather than proof of, our choice of the rule-based route here. The U-Net baseline remains relevant for future extensions to anatomies with greater morphological variability and for incorporating image-driven feedback into the control loop.

### 4.8. Volunteer Feedback

**Figure 11:**
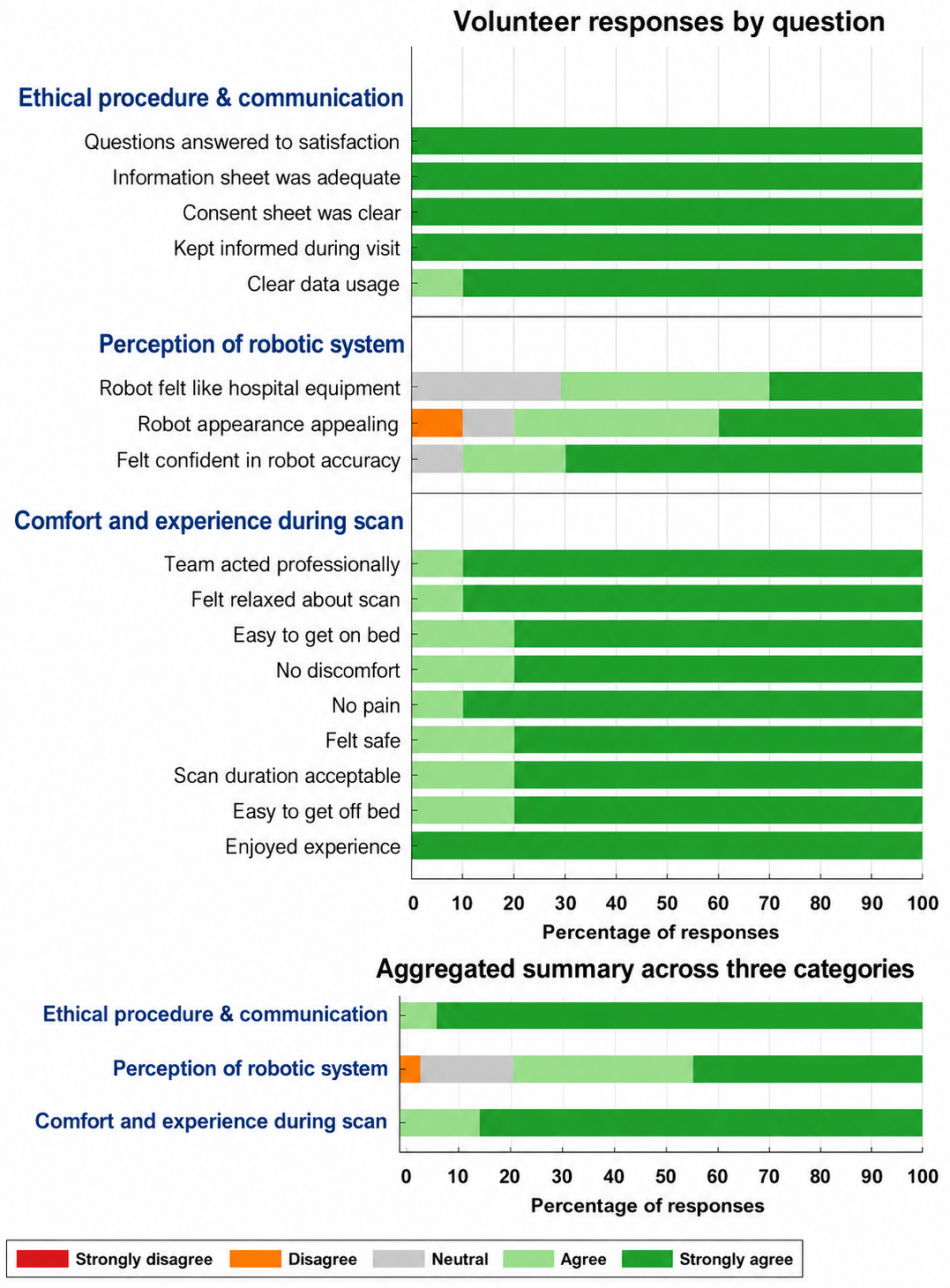
Volunteer responses to seventeen questionnaire items grouped by three dimensions (ethical procedure and communication; perception of the robotic system; and comfort and experience during the scan), with an aggregated summary across the three categories showing the overall level of agreement. The legend shows the complete five-point Likert scale presented to volunteers; “Strongly disagree” (red) is retained for completeness but does not appear in any bar because it was never selected for any item.

Post-scan questionnaires showed consistently positive responses across all categories (Figure 11). For ethical procedure and communication, agreement was virtually unanimous. Participants strongly agreed that the information sheet and consent form were clear (100%), that they were kept informed throughout (100%), and that their questions were answered satisfactorily (100%). Clarity of data usage was also rated highly, with 90% ‘strongly agree’ and 10% ‘agree’.

For perception of the robotic system, responses were more varied. Confidence in the robot’s accuracy was high (70% ‘strongly agree’, 20% ‘agree’), but only 70% felt it resembled hospital equipment, and 20% rated its appearance as neutral or unappealing.

For comfort and experience, feedback was unanimously positive. All volunteers reported no pain or discomfort (100% positive), felt safe throughout the procedure (80% ‘strongly agree’, 20% ‘agree’), and considered the scan duration acceptable (80%/20%).

Overall, acceptance of the protocol was high. Ethical- and comfort-related items were uniformly positive, while perception-related items were also favourable (90% positive overall) and suggested modest scope for refinement, particularly in improving the robot’s external appearance. Importantly, no concerns were raised regarding the clarity of data-usage information.

## 5. Future Work

While this feasibility study demonstrates that operator-supervised, force-controlled autonomous sweeps combined with offline image analysis can produce diagnostically usable aortic images in all volunteers, several steps remain before translation to routine screening. The contribution of this work lies in integrating patient-to-robot registration, surface-following path planning, hybrid position–force control with a low-cost force sensor, rule-based image-quality assessment, and automated diameter estimation into a single, low-cost, end-to-end pipeline, and in validating it against clinician measurements in human subjects; to our knowledge, this combination and in vivo validation have not previously been reported for autonomous AAA screening. Building each component on proven, transparent methods is a deliberate design choice that favours robustness, interpretability, and deployability. Clinically, our cohort is small and demographically narrow: ten healthy volunteers from a single centre, nine of whom were aged 20–30 with only one participant in the 50–60 age band most relevant to AAA screening, and with no confirmed aneurysm, intraluminal thrombus, or other vascular pathology. The screening claim is therefore one of feasibility (that the system can acquire diagnostic images and produce automated diameter estimates in a young, healthy population) rather than of validated diagnostic performance. Future studies should include larger, multi-centre cohorts that explicitly recruit the older and higher-BMI screening population, and patients with established aneurysms (including thrombus) and irregular morphology; the geometric assumptions of the current diameter-estimation pipeline (Section 4.6) will then require relaxation, and a learning-based segmentation route (Section 3.3) may become preferable to the rule-based pipeline used here. We are planning further volunteer studies with a larger, prospectively powered cohort; beyond broadening the demographic range, this would allow the exploratory sex-, BMI-, and body-composition trends reported here to be tested with adequate statistical power, rather than remaining the qualitative, hypothesis-generating observations they necessarily are with only ten volunteers.

Technically, the current image-analysis pipeline runs entirely post-acquisition: there is no real-time closed-loop control linking image content back to the sweep. As a consequence, the robot cannot autonomously revisit low-quality regions, retract from a poor acoustic window, or terminate early once sufficient diagnostic views are obtained, and instead relies on the operator to repeat the sweep when needed. A key next step is to integrate QA, aorta localisation, and diameter estimation directly into the control loop, similar to image-driven Bayesian-optimisation approaches [38,39], to enable adaptive scanning, reduce examination time, and shrink the operator-in-the-loop steps that still bound the autonomy of the present system.

In parallel, as larger labelled datasets and computational resources become available, we will investigate combining the existing rule-based analysis with learned components (including the U-Net branch in Section 3.3) to support more challenging applications where anatomy is smaller, motion is faster, or acoustic windows are less predictable, while prioritising computational practicality and sufficient transparency for clinical trust and safety. Two specific evaluations follow directly from the present limitations: (i) an end-to-end diameter error computed from the U-Net’s own predicted masks (rather than the ground-truth-mask oracle of Table 4), on a subject-held-out cohort large enough to test cross-subject generalisation; and (ii) a dedicated high-BMI/obese study to determine whether the 15 N safety cap is sufficient for the deep acoustic windows that manual practice addresses with much higher forces, together with the penetration-preserving mitigations discussed in Section 4.

Beyond AAA screening, the large workspace of the dual-arm platform and its top-down, anterior surface-following scanning make it well suited to general abdominal ultrasound. The most natural extensions therefore lie within the abdomen: obstetric and fetal scanning (the platform was in fact originally developed for fetal screening [41]), hepatic (liver) imaging, renal and gallbladder or biliary assessment, spleen and pancreas surveys, bladder-volume estimation, and focused abdominal assessment such as free-fluid (FAST) screening. The same building blocks (RGB-depth patient-to-robot registration, low-cost force-controlled surface-following sweeps, and a transparent, training-free image-analysis pipeline) are largely anatomy-agnostic and could carry over to these targets with task-specific localisation rules or a learned segmenter and QA weights re-tuned against site-specific clinician labels. More broadly, the force-driven design, which avoids dependence on proprietary real-time image interfaces, may be attractive for deployment in resource-limited or point-of-care settings and for standardised, repeatable follow-up imaging.

## 6. Conclusions

This work presented a conditionally autonomous (Level-3) robotic ultrasound system for abdominal aortic aneurysm screening, combining RGB-D-based patient-to-robot registration, surface-constrained path planning, hybrid position–force control, automatic diagnostic-frame selection, and rule-based aortic diameter estimation in a single end-to-end pipeline. Operator involvement is confined to defining the scan region and confirming the target force band; the subsequent sweep, frame ranking, and diameter estimation are performed without further manual probe manipulation. In a feasibility study on ten healthy volunteers (BMI 18.6–33; mixed sex and skin tone; nine aged 20–30 and one aged 50–60), the system consistently acquired clinician-verified diagnostic aortic views under conservative force limits, achieved automated diameter measurements generally within accepted screening tolerances, and was well accepted by participants. These results suggest that autonomous robotic sweeps can be compatible with existing clinical workflows while reducing reliance on manual probe manipulation and subjective frame selection, and they provide a foundation for future work on larger, more diverse cohorts and tighter real-time integration of image feedback into control.

## Figures and Tables

**Table 1:** Volunteer demographics.

ID	Gender	BMI	Age
1	Male	26	20–30
2	Male	33	50–60
3	Male	19.6	20–30
4	Male	21.6	20–30
5	Male	24.2	20–30
6	Male	20	20–30
7	Male	23.8	20–30
8	Female	26	20–30
9	Female	19.9	20–30
10	Female	18.6	20–30

**Table 2:** Rule-based abdominal aortic diameter estimation vs clinician on-machine measurements.

Volunteer ID	Calculated Diameter (mm)	Actual Diameter (mm)	Difference (mm)
1	13.2	15.6	−2.4
2	17.8	17.9	−0.1
3	14.2	13.4	0.8
4	13.9	13.4	0.5
5	11.6	16.2	−4.6
6	13.1	14.9	−1.8
7	16.8	15.7	1.1
8	11.6	12.7	−1.1
9	13.3	12.5	0.8
10	13.0	11.7	1.3

**Table 3:** U-Net segmentation performance on n=10 real-ultrasound test frames (Stage 3 final model).

Metric	Mean	±Std
Dice coefficient	0.921	±0.033
IoU (Jaccard index)	0.856	±0.056

**Table 4:** Depth-corrected diameter validation computed from clinician ground-truth (GT) masks (an oracle/upper-bound estimate, *not* from U-Net predictions) versus clinician on-machine measurements. “Calculated” is the maximum-inscribed-circle diameter on the GT mask, averaged over the “# Frames” annotated frames for each subject.

Volunteer ID	# Frames	Calculated (mm)	GT (mm)	Err. (mm)
1	8	13.9 ± 2.9	15.6	−1.7
2	15	20.0 ± 8.0	17.9	+2.1
3	5	14.4 ± 0.5	13.4	+1.0
4	3	17.4 ± 6.1	13.4	+4.0
5	4	14.7 ± 1.1	16.2	−1.5
6	14	13.7 ± 0.6	14.9	−1.2
7	8	14.3 ± 1.5	15.7	−1.4
8	13	12.1 ± 0.8	12.7	−0.6
9	11	12.9 ± 0.7	12.5	+0.4
10	11	11.8 ± 0.7	11.7	+0.1

## Data Availability

The data presented in this study are available on reasonable request from the corresponding author. The data are not publicly available due to ethics restrictions concerning volunteer privacy.
